# The return of the Beaker Folk? Rethinking migration and population change in British prehistory

**DOI:** 10.15184/aqy.2021.129

**Published:** 2021-08-31

**Authors:** Ian Armit, David Reich

**Affiliations:** 1 Department of Archaeology, University of York, King’s Manor, Exhibition Square, York YO1 7EP; 2 Department of Genetics, Harvard Medical School, Boston, Massachusetts 02115, USA; 3 Department of Human Evolutionary Biology, Harvard University, Cambridge, MA 02138, USA; 4 Broad Institute of Harvard and MIT, Cambridge, MA, 02142, USA; 5 Howard Hughes Medical Institute, Harvard Medical School, Boston, MA 02115, USA

**Keywords:** Beaker, migration, Neolithic, Bronze Age, Chalcolithic, aDNA

## Abstract

Recent aDNA analysis has demonstrated that the centuries surrounding the arrival of the Beaker Complex in Britain witnessed a massive turnover in the genetic make-up of the population. Here we consider the archaeological implications of this finding, and propose two hypotheses - Beaker Colonisation and Steppe Drift - that might help us understand the underlying social processes, and propose directions for future research.

## The Beaker Complex in Britain and Europe

One of the major marker points in British prehistory is the appearance of Beaker pottery in the mid-third millennium BC (FIG 1), occurring as part of a ‘cultural package’ characterised by single inhumation burials, Continental-style archery equipment and objects of gold and copper. Based on the available radiocarbon dates, the ‘Beaker Complex’ is often viewed as having originated in Iberia around 2750 BC (Cardoso 2014), from where it spread rapidly across much of Europe and north-west Africa (FIG 2), arriving in Britain from around 2450 BC (Jay et al. 2019: 75).

For most of the twentieth century, the spread of the Beaker Complex was interpreted as the product of migration. Although founded principally on the marked changes evident in material culture and burial practices (not least the form and decoration of Beaker vessels themselves), this hypothesis was strengthened in Britain by the observation of a shift in the average cranial shape in burial populations: from the relatively more dolichocephalic (long-headed) shape of Neolithic populations, to the relatively more brachycephalic (round-headed) individuals associated with Beakers (FIG 3).

During the 1960s scepticism began to grow about the primacy of migration as a vector of social change in prehistory. Nonetheless, even in Grahame Clark’s classic statement on the inadequacy of models founded on putative invasions, the intrusive nature of the Beaker Complex remained essentially unquestioned (Clark 1966: 160). It was only in the mid-1970s, with the consolidation of processual approaches, that alternative explanations began to gain traction. Foremost among these was the suggestion that the Beaker Complex related to the spread of a prestige set of objects, practices and beliefs among the upper echelons of society at the start of the European Bronze Age (e.g. Burgess and Shennan 1976). The uptake of this new elite cultural package was suggested to have been promoted by its association with the prestigious new metal, and/or with the spread of religious behaviours involving the consumption of alcohol (e.g. Sherratt 1987). Even the change in skull shape associated with the appearance of Beaker pottery in Britain could potentially be accounted for as the result of deliberate or accidental cranial shaping (Parker Pearson et al. 2016: 625). Nonetheless, the most detailed and authoritative studies on the Beaker Complex in Britain have held out the possibility of some level of migration from the Continent, even if restricted to relatively small numbers (e.g. Needham 2005).

The recent emergence of genome-wide analysis of ancient DNA provides, for the first time, a means to investigate the question of migration directly, at the population scale, through the genetic signatures of the individuals buried with Beaker Complex artefacts. Unlike osteological or isotopic analysis, these genetic data provide information not only on the individuals themselves, but on the ancestral populations from which they derive.

## The new genetic data

A recent study involving the present authors presented genome-wide data from 400 individuals spanning the Neolithic to the Middle Bronze Age, including 226 associated with distinctive artefacts belonging to the Beaker Complex (Olalde et al. 2018). This work produced two central conclusions relevant to the mechanisms by which the Beaker Complex spread.

Firstly, the study demonstrated that while Beaker Complex individuals in Iberia derived most of their ancestry from local, Neolithic farming groups, those in Central Europe had a quite different genetic profile. Earlier aDNA analysis had demonstrated that the centuries from around 3000–2600 BC witnessed the spread into northern and Central Europe of populations most of whose ancestry derived ultimately from Early Bronze Age pastoralists associated with the Yamnaya cultures of the Eurasian steppe (e.g. Haak et al. 2015; Allentoft et al. 2015). By the end of the Neolithic, all sampled populations in these areas (Corded Ware Culture) had substantial proportions of steppe ancestry, although there was considerable local variation in the precise amount. It was these communities who adopted the Beaker Complex in Central Europe. This contrast between Iberian and Central European Beaker Complex communities is exemplified by the Y-chromosome data: those in Iberia comprise Y-chromosome lineages common since the start of the Neolithic in that region, while those in Central Europe are dominated by a single lineage (RIb-M269) derived from the steppe (Olalde et al. 2018). The cultural traits associated with the Beaker phenomenon in Continental Europe did not, therefore, spread principally through migration, but must have involved the movement of ideas between populations of distinct genetic heritage.

It is the study’s second main conclusion, however, that is most relevant here. Whereas Neolithic individuals from Britain (n=51) lack any trace of Continental/steppe ancestry, all those associated with Beaker Complex artefacts (n=37), and all those from the subsequent Early and Middle Bronze Ages (n=67), display large amounts (FIG. 4). More than 90% of these Beaker Complex and later male individuals, for example, belong to Y-chromosome haplogroup RIb, previously absent in Neolithic Britain but a definitive indicator of steppe ancestry common in Central Europe, demonstrating the arrival of substantial numbers of men from the Continent. Mitochondrial haplogroups not previously present in Britain (e.g. I, R1a and U4) show that women also moved in substantial numbers at this time. Thus, while genetic data do not preclude some degree of male-bias in the westward spread of Steppe ancestry (e.g. Kristiansen 2017), at least in Britain we can rule out scenarios of male-only Steppe ancestry spread. The genetic evidence, assessed at the level of the whole genome, shows a replacement of 93% of the gene pool in Britain, suggesting that the arrival of the Beaker Complex correlates with a massive turnover of population during the last centuries of the third millennium BC.

Although the aDNA data cannot presently demonstrate the source area for this influx of new people (geographical gaps in the sample affect potential key areas - notably the northern coastal regions of France, Belgium and Germany), Beaker Complex individuals from Britain are genetically closest to those from the Lower Rhine area, and particularly to individuals from the site of Oostwoud in the Netherlands (Olalde et al. 2018). This genetic link accords well with the ceramic evidence that shows close stylistic relationships (e.g. the dominance of All-Over-Corded Beakers in both regions) between the Netherlands and Britain, suggestive of at least one major source population originating somewhere in that broad region. The archaeological record for potentially relevant areas of the Continent is not yet sufficiently refined to enable the identification of any diminution in regional populations corresponding to the movement of people into Britain; indeed, if population pressure formed part of the motivation for population movement, then we might expect the populations of the source areas to remain relatively stable overall.

## Reactions and objections

It is no overstatement to suggest that the new aDNA data dramatically transform our understanding of British prehistory. Indeed, the recent genetic study has been characterised as a “bombshell” thrown into the ongoing debates around Beaker origins (Ray and Thomas 2018: 279). In essence, the results demonstrate that for the typical individual living in Britain at the end of the third millennium (or at least for all those sampled so far), more than 90% of their ancestors living c. 2500 BC would have been resident in Continental Europe (Olalde et al. 2018). The indigenous Neolithic populations of Britain, who in turn derived the vast majority of their ancestry from earlier migrants (Brace et al. 2019), made only a small genetic contribution to those of the Bronze Age. This not only confirms the central role of population movement in the transmission of the Beaker Complex into Britain, but also demonstrates a scale of migration that was wholly unanticipated in earlier debates: a virtual ground zero for the prehistoric settlement history of Britain.

There have been objections to this interpretation. Ray and Thomas, for example, have questioned the validity of conclusions drawn from the analysis of what they regard as “few and fragmentary ancient DNA samples” (2018: 280). This suggestion is, however, based on a misunderstanding on the nature of aDNA evidence. Unlike osteological and isotope analysis, aDNA data document the genetic inheritance not just of the sampled individual, but also of their ancestors (Li & Durbin 2011). Each of the 37 Beaker Complex individuals sampled in Britain represents, in effect, a human population in miniature (cf. Booth 2019, 588). While an increased density of sample coverage would undoubtedly be highly beneficial (see below), the sample analysed by Olalde et al. (2018) is substantial and robust.

A more significant objection is the question of sampling bias. It could be argued, for example, that the archaeological visibility of Beaker Complex burials, and the long-standing archaeological interest in their associated material culture, has led to an over-sampling of precisely those individuals most likely to represent migrants from the Continent. Perhaps, it might be argued, the population turnover associated with the Beaker Complex was actually limited to a small number of possibly high-status incomers, whose prominence in death has exaggerated their importance as a component of the population of the time. There is, however, a counter-argument. While it may be true (indeed it is highly likely) that aDNA sampling for the period c. 2500–2100 BC has focused disproportionately on those individuals buried with Beaker Complex grave associations, the overwhelming dominance of steppe ancestry in *subsequent* populations (i.e. those of the Middle and Late Bronze Age) demonstrates that these incomers were sufficiently numerous, at least in relative terms, to initiate a massive genetic turnover. If this were not so, one would expect to see a re-emergence of the earlier genetic picture, as these Beaker Complex migrants were gradually absorbed into the indigenous population over the ensuing centuries. As can be seen from FIG. 4, however, this does not happen. Nor can it simply be the case that a minority of high-status male incomers had a disproportionate number of offspring, since we know that Beaker Complex individuals included both men and women from the Continent, introducing new dominant Y-chromosome and mitochondrial lineages. While the absolute scale of migration is unknown, the incomers must have been sufficiently numerous to demographically overwhelm the existing population within the space of a few centuries.

At first glance, the genetic results might seem to contradict a recent large-scale multi-isotope analysis of large numbers of individuals associated with Beaker Complex artefacts, which identified considerable evidence for individual mobility at a relatively local scale, but nothing to suggest substantial cross-Channel movement of people into Britain (Parker Pearson et al. 2019). Individual migration from the Continent had been suggested for a few individuals, including the well-known Amesbury Archer, recovered from an unusually richly-outfitted Beaker Complex grave close to Stonehenge, dated to 2380–2290 cal BC (Barclay et al. 2011), but was judged atypical overall. This apparent contradiction is, however, easily resolved. As acknowledged by Parker Pearson et al. (2019: 437), isotopic analysis deals with the life course of the specific individual under study and can thus at best identify only first-generation migrants. Indeed, even this can be highly problematic where the isotopic profile of the two regions is similar, as it would be for migrants moving from the chalklands of northern France or Belgium into southern Britain. A putative son or daughter of Continental immigrants, raised on the Wessex chalklands, will appear wholly local from an isotopic perspective. Ancient DNA, by contrast, would identify that individual as deriving from a Continental population. The two forms of analysis thus give quite different (yet complementary) insights into population history. We will return to the importance of this isotopic analysis below.

## Late Neolithic population levels

The scale of the genetic transformation associated with the arrival of the Beaker Complex has significant implications for our understanding of Late Neolithic population levels in Britain. If large numbers of Beaker Complex migrants had encountered populous Late Neolithic landscapes, we might expect some evidence for conflict. Yet, while there is much osteological evidence for lethal violence within earlier Neolithic communities (e.g. Schulting & Wysocki 2005), there is little that can be attributed to the period under consideration here. While it is always difficult to document levels of inter-personal violence through archaeological evidence (e.g. Armit 2011), the apparent absence of mass graves, battle or skirmish sites, or any increase in the incidence of violent injury, provides no evidence of large-scale violent invasion or confrontational migratory movements. Is it possible then that the indigenous, Late Neolithic population of Britain was sufficiently small and/or sparsely-distributed that large groups of incomers could be accommodated without substantial conflict over resources?

There is in fact mounting evidence that the indigenous Neolithic populations of Britain may indeed have been relatively low in the centuries leading up to the arrival of the Beaker Complex. Analysis of summed radiocarbon probability distributions for burnt cereal grains, for example, appears to show a sharp drop in agricultural activity in mainland Britain at the start of the Middle Neolithic, c. 3350 BC, and a further decline at c. 2850 BC, ushering in a remarkably low level of arable cultivation that persists until c. 2450 BC (Stevens & Fuller 2012; 2015, fig. 1). The subsequent recovery corresponds to the period at which the first indications of the Beaker Complex in Britain occur.

Although these summed probability distributions have not been modelled to take account of variations in the radiocarbon calibration curve (cf. Armit et al. 2013, 2014), the broad picture is that there is little evidence of agricultural activity in Britain from 2900 to 2450 BC. This finding is supported by other strands of evidence, including a low incidence of dental caries associated with cereal consumption (McKinley 2008), isotope evidence for an increased reliance on animal protein (Richards 2000), indications of woodland regeneration (Shennan et al. 2013), soil degradation (Mills et al. 2014), an apparent cessation of contacts between insular communities and those of Continental Europe (Carlin 2018, 198; Wilkin & Vander Linden 2015, 104), and a general paucity of settlement evidence. All of this adds weight to interpretations of a general demographic decline across Britain at this time. The causes may have been many and complex: climatic factors have been suggested (Stevens and Fuller 2015: 867); over-exploitation of agricultural soils clearly played a part in some areas (Mills et al. 2014); and exposure to new pathogens, perhaps introduced by the immigrant populations, may also have been a factor (cf. Valtueña et al. 2017; Rascovan et al. 2019). The resultant availability of under-utilised land may well have been a significant pull-factor, encouraging the movement of people from the near Continent. Indeed, isotope analysis suggests significant internal mobility within Britain from c. 2400 – 2100 BC (Parker Pearson et al. 2019), consistent with the fissioning of recently-arrived migrant communities over a few generations to occupy available pockets of farmland as their populations expanded.

This evidence for population decline in Late Neolithic Britain mirrors a broader picture across much of temperate Europe (e.g. Shennan et al. 2013; Kristiansen 2015). In a British context, it is especially interesting given the manifest continuity of monument construction, which might be taken at face value to suggest the presence of large settled populations. Recent analysis of faunal remains from four major monument complexes in Wessex, however, suggests that pigs were imported to these sites from multiple locations over remarkably long distances (Madgwick et al. 2019), adding to previous work that suggested the presence of non-local cattle at Durrington Walls (Viner et al. 2010). This accumulating evidence suggests that people from across many regions of Britain were potentially implicated in the construction and use of such monuments. Indeed, rather than representing a cultural floruit of populous regional chiefdoms, these ambitious construction projects may instead reflect cultural responses to a period of existential crisis for Neolithic communities, drawing on extensive networks of people from small communities dispersed over wide regions.

## Discussion

It has been a common critique of aDNA studies that they have conflated genetic with ethnic identities (e.g. Vander Linden 2016; Frieman & Hofmann 2019). With this in mind it is important to point out that, although the earliest individuals in Britain who can be identified as having steppe ancestry are associated with the Beaker Complex, this need not signal some folk movement of the type conceived by early twentieth century diffusionists such as Kossinna (cf. Heyd 2017). Indeed, while the chronological correspondence between population turnover and the arrival of the Beaker Complex in Britain is striking, the true nature of the relationship between them remains to be established. This is particularly important because, as we have seen in the rest of Europe, there is no consistent connection between the spread of the Beaker Complex and changes in the genetic make-up of a population (Olalde et al. 2018).

Rather than immediately labelling the transformations that affected Britain as a ‘coming of the Beaker Folk’, we need instead to examine closely the relationship between, on the one hand, the extension into Britain of a broader, Continent-wide spread of steppe ancestry, deriving ultimately from the Yamnaya or their close relatives on the steppe; and on the other, the appearance of the objects and practices that comprise the Beaker Complex. Since the first of these phenomena is genetic and the second cultural, we must be careful not to conflate them. Indeed, we can perhaps frame their relationship by stating two competing hypotheses:

1. The Beaker Colonisation hypothesis: steppe ancestry was introduced into Britain through the arrival of migrants associated with the Beaker Complex over a period of a no more than a few centuries from c. 2450–2100 BC (and perhaps over a much shorter period). The migration event(s) are intimately bound up with the expansion of Beaker cultural practices and values: the documented genetic and cultural changes affecting Britain at this time are thus intimately linked.
2. The Steppe Drift hypothesis: steppe ancestry was introduced to Britain as an extension of its general westward movement across Europe, through a gradual process beginning perhaps as early as 2700 BC (when it appears in Central Europe) and ending as late as 2000 BC. The parallel north and eastward movement of the Beaker Complex from Iberia was an essentially cultural phenomenon with no intrinsic genetic component. Due to the intersection of these genetic and cultural phenomena, migrants into Britain from around 2450 BC frequently came equipped with objects and ideas associated with the Beaker Complex. Steppe ancestry in Britain becomes detectable with the emergence of this highly archaeologically-visible burial rite, but the genetic and cultural changes represent the collision of two separate, Continent-wide processes.

In order to differentiate between these hypotheses, we need to gather targeted data from the critical period c. 2600–2000 BC to establish whether we can see a hard line in the presence/absence of steppe ancestry between the latest Neolithic populations and those of the Early Bronze Age. This is not, however, straightforward. Funerary practice across Late Neolithic Britain is extremely fugitive and excavated human remains are dominated by cremation, with the largest known cemetery being at Stonehenge (e.g. Willis et al. 2016: 352–3). Aside from a small number of individuals in Orcadian chambered tombs (Armit et al. 2016), inhumations are virtually absent in the critical centuries prior to the appearance of the Beaker Complex. Indeed, most individuals during this period were probably subject to mortuary practices (such as excarnation by exposure) that leave little archaeological trace. As a result, few samples are available for aDNA analysis. Had steppe ancestry been introduced before the arrival of the Beaker Complex, it would thus be extremely hard to detect.

In addition to the problem of identifying the earliest individuals in Britain with steppe ancestry, it is equally hard to find the latest individuals that lack it. There are at least two ways in which the current aDNA sample may underestimate the survival of indigenous populations. The first concerns the uneven spatial distribution of the available aDNA sample which, although it ranges from south-east England to the Western Isles of Scotland, nonetheless contains geographical gaps (e.g. north-east Scotland, East Anglia). Conceivably, regional pockets of population lacking steppe ancestry may have survived for several centuries in such areas. Secondly, indigenous groups in more densely-sampled areas may appear absent because they did not practice inhumation burial (cf. Parker Pearson et al. 2019). This possibility is strengthened by recent aDNA analysis in Iberia that shows apparently distinct populations (with and without steppe ancestry) surviving side-by-side for several centuries in the late third millennium BC (Olalde et al. 2019). The aDNA evidence does not, therefore, preclude the survival of indigenous populations in the centuries following the first appearance of the Beaker Complex in Britain.

The more gradual arrival of individuals bearing steppe ancestry, together with the survival of some indigenous populations in the last few centuries of the third millennium BC (as predicted by the Steppe Drift hypothesis) may help to explain the otherwise puzzling continuity seen in much of the archaeological record over the second half of the third millennium BC. While the settlement record for Late Neolithic Britain is undoubtedly sparse, we have already noted that this was a period of monumental construction on a grand scale. Indeed, several major monument complexes continued to be elaborated and remodelled in the critical centuries of the late third millennium BC. Stage 3 at Stonehenge, for example, which involved the re-cutting of the main earthwork ditch and the construction of the Avenue, appears to have been associated with Beaker Complex inhumations (Darvill et al. 2012), while the nearby burial of the Amesbury Archer demonstrates the continuing importance of the wider Stonehenge landscape to incoming peoples. The monumental landscape around Avebury too, continued to develop through this period. Indeed, the construction of the massive mound of Silbury Hill, c. 2470 to 2350 BC (Leary et al. 2014), appears to belong in its entirety to the period of initial Beaker Complex incursions.

There is one further strand of evidence to support the idea that some indigenous populations survived through the Early Bronze Age. While several Beaker Complex individuals in the aDNA sample appear to be first generation migrants (or at least wholly descended from Continental communities), most have some ancestry derived from indigenous populations and indeed some outlier individuals have substantial proportions (FIG 4), suggesting mixing between these groups from an early stage and persistence of individuals with high proportions of Neolithic-derived ancestry for a couple of hundred years following the arrival of Beaker Complex traditions (even if they are not directly sampled in available ancient DNA data). By the Middle Bronze Age, the median proportion of Neolithic-associated ancestry increased and there was less variability in Neolithic ancestry across individuals (FIG 4), showing how persistent indigenous groups and the migrants from the Continent had thoroughly mixed by this time.

## Conclusions

In the wake of the aDNA results, there can be no doubt that population movement into Britain from the Continent was on a scale sufficient to produce a genetic turnover equating to more than 90% between c. 2500–2000 BC. Although this finding represents a major advance in our understanding of the population history of prehistoric Britain, it does not provide an explanation for the phenomenon in social and cultural terms. Instead the new genetic data opens up a range of possible scenarios which are potentially testable through future archaeological and aDNA analyses.

We have framed two competing hypotheses that might explain the genetic changes. If the Beaker Colonisation hypothesis is correct, future DNA analysis can be expected to reinforce a clear genetic disjunction between those associated with Beaker Complex burial rites and those who are not; it should further indicate a sudden and widespread appearance of steppe ancestry in Britain from around 2450 BC. By contrast, if the Steppe Drift hypothesis is correct, future work should reveal indications of steppe ancestry in some culturally and/or chronologically Late Neolithic individuals. Inhumations dating to the period from around 2600–2200 BC are thus at an absolute premium for aDNA analysis.

A critical difference between the two hypotheses lies in the perceived relationship between objects and genes: ethnic and biological identities. Were the Continental migrants who arrived in Britain in the mid-third millennium BC conscious of their part in a cultural (and genetic) takeover, imposing new beliefs and practices on an established population? Or was the incoming movement of new people more gradual, fragmented, and independent of wider cultural change? These questions are capable of resolution, partly through future aDNA analysis if appropriate samples from the key time periods and geographical regions become available. But they will require an open mindedness amongst archaeologists to develop and test new models that take full account of the genetic data.

## Figures and Tables

**1.**
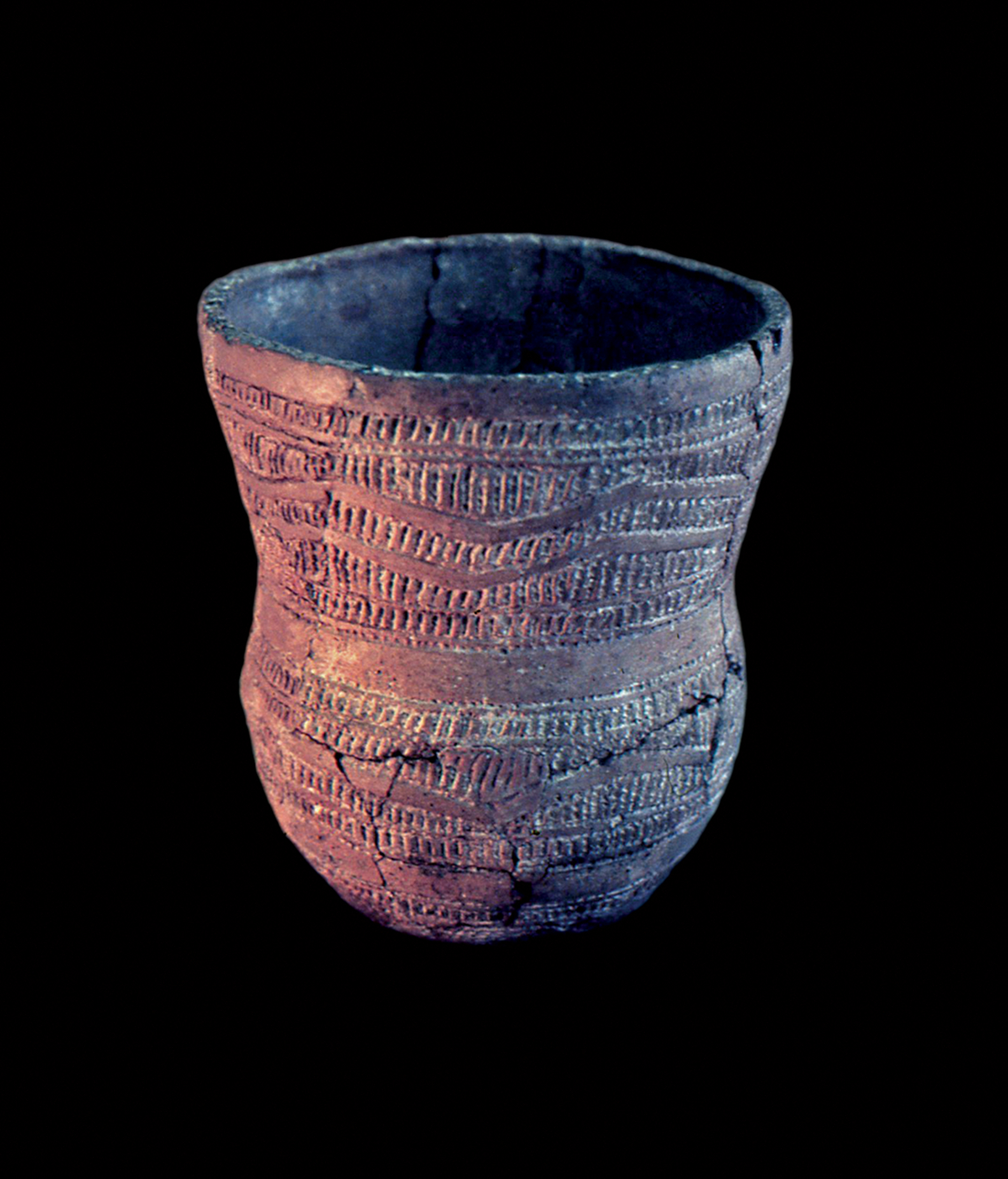
Beaker vessel from Wetwang Slack, East Yorkshire (Wetwang/Garton Slack archive).

**2.**
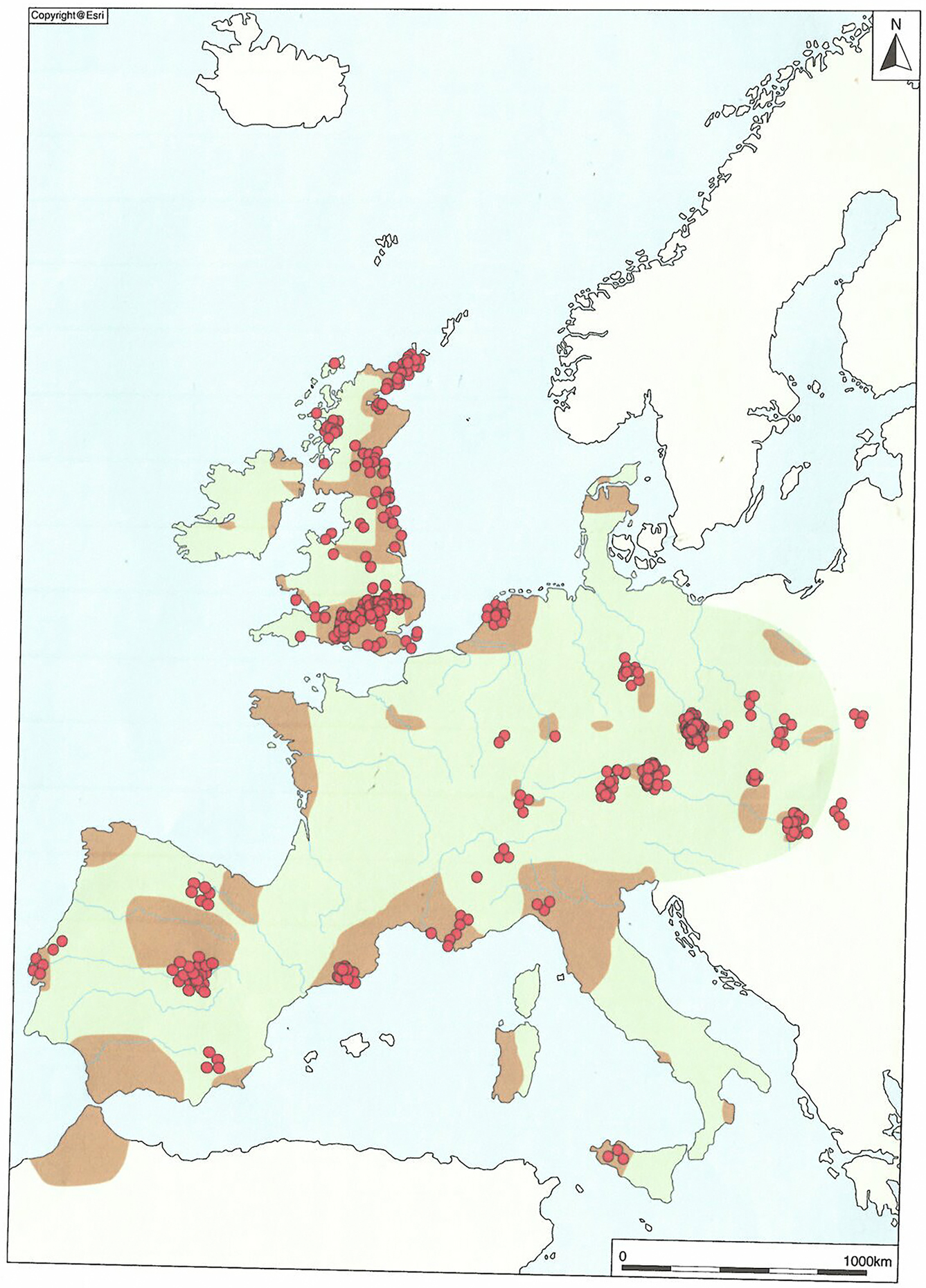
The Beaker Complex in Europe, with red dots indicating the locations of individuals sampled for aDNA.

**3.**
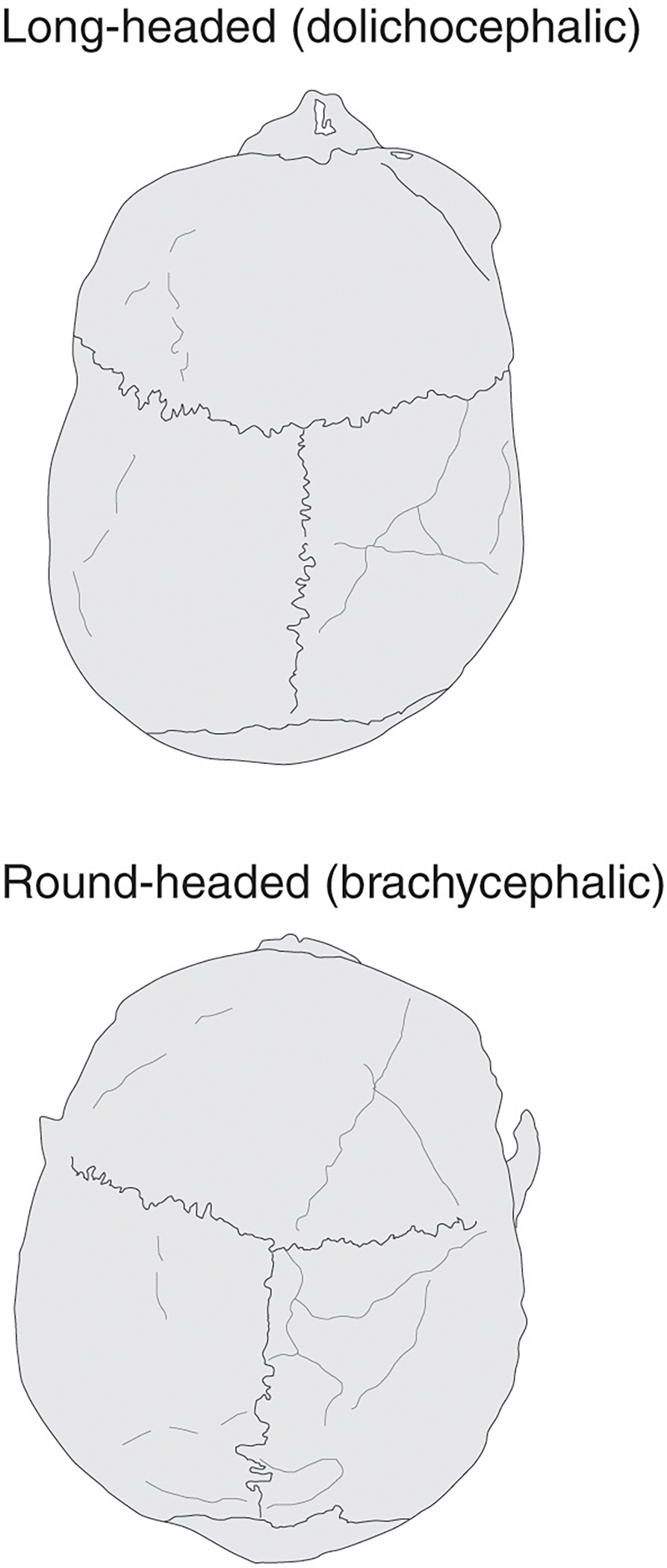
Long-headed (*dolichocephalic*) individuals were associated with Neolithic chambered tombs and long barrows, while round-headed (*brachycephalic*) skulls characterise with the Beaker Complex.

**4.**
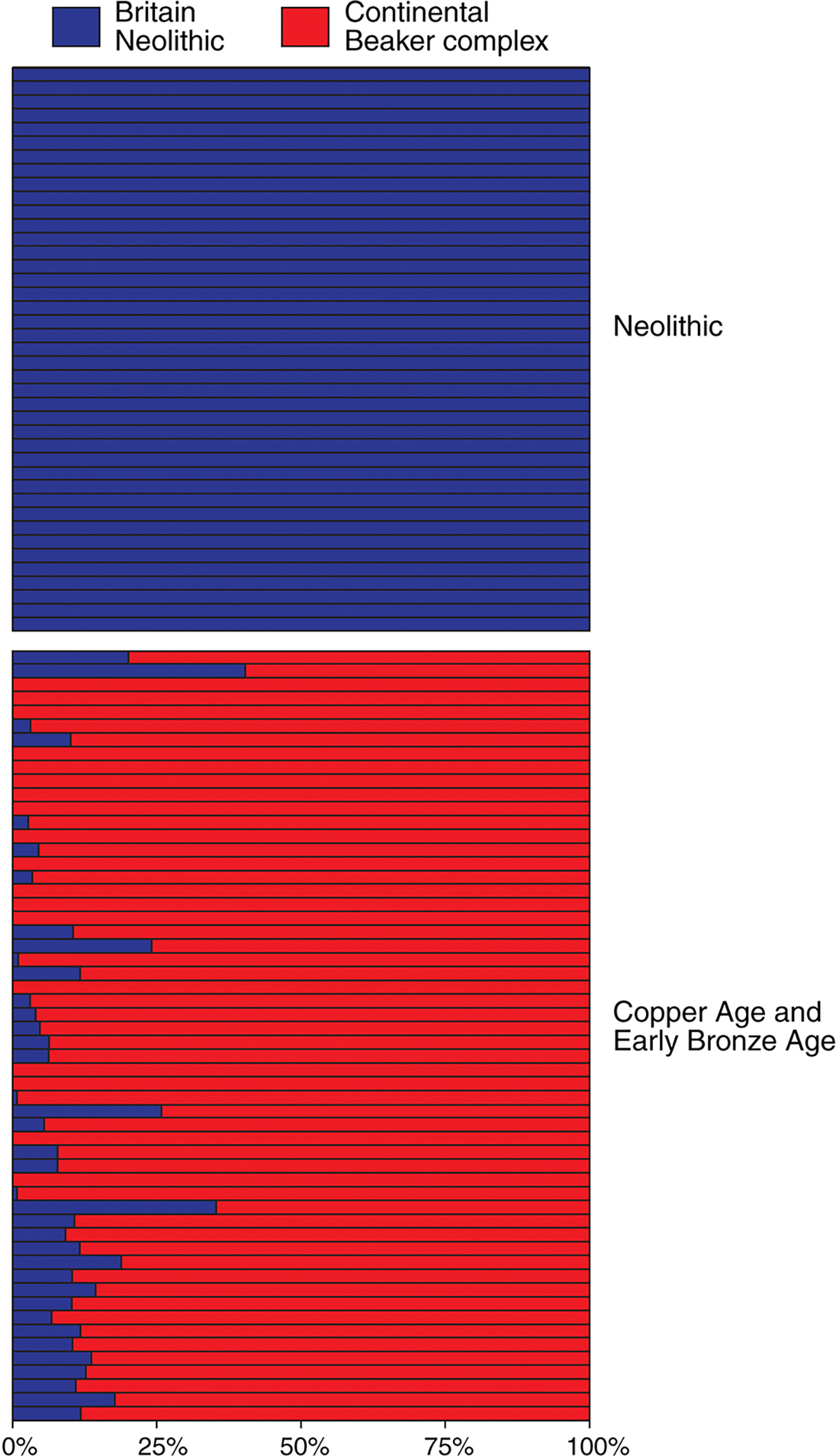
Proportions of continental/steppe (red) and British Neolithic (blue) ancestry in Neolithic, Chalcolithic and Early Bronze Age individuals from Britain sampled as part of the recent aDNA study. Each horizontal bar represents one individual, ordered from the earliest (top) to most recent (bottom). The underlying data derive from Olalde et al. 2018.
